# Implicit bias in healthcare professionals: a systematic review

**DOI:** 10.1186/s12910-017-0179-8

**Published:** 2017-03-01

**Authors:** Chloë FitzGerald, Samia Hurst

**Affiliations:** Institute for Ethics, History, and the Humanities, Faculty of Medicine University of Geneva, Genève, Switzerland

**Keywords:** Implicit bias, Prejudice, Stereotyping, Attitudes of health personnel, Healthcare disparities

## Abstract

**Background:**

Implicit biases involve associations outside conscious awareness that lead to a negative evaluation of a person on the basis of irrelevant characteristics such as race or gender. This review examines the evidence that healthcare professionals display implicit biases towards patients.

**Methods:**

PubMed, PsychINFO, PsychARTICLE and CINAHL were searched for peer-reviewed articles published between 1st March 2003 and 31st March 2013. Two reviewers assessed the eligibility of the identified papers based on precise content and quality criteria. The references of eligible papers were examined to identify further eligible studies.

**Results:**

Forty two articles were identified as eligible. Seventeen used an implicit measure (Implicit Association Test in fifteen and subliminal priming in two), to test the biases of healthcare professionals. Twenty five articles employed a between-subjects design, using vignettes to examine the influence of patient characteristics on healthcare professionals’ attitudes, diagnoses, and treatment decisions. The second method was included although it does not isolate implicit attitudes because it is recognised by psychologists who specialise in implicit cognition as a way of detecting the possible presence of implicit bias. Twenty seven studies examined racial/ethnic biases; ten other biases were investigated, including gender, age and weight. Thirty five articles found evidence of implicit bias in healthcare professionals; all the studies that investigated correlations found a significant positive relationship between level of implicit bias and lower quality of care.

**Discussion:**

The evidence indicates that healthcare professionals exhibit the same levels of implicit bias as the wider population. The interactions between multiple patient characteristics and between healthcare professional and patient characteristics reveal the complexity of the phenomenon of implicit bias and its influence on clinician-patient interaction. The most convincing studies from our review are those that combine the IAT and a method measuring the quality of treatment in the actual world. Correlational evidence indicates that biases are likely to influence diagnosis and treatment decisions and levels of care in some circumstances and need to be further investigated. Our review also indicates that there may sometimes be a gap between the norm of impartiality and the extent to which it is embraced by healthcare professionals for some of the tested characteristics.

**Conclusions:**

Our findings highlight the need for the healthcare profession to address the role of implicit biases in disparities in healthcare. More research in actual care settings and a greater homogeneity in methods employed to test implicit biases in healthcare is needed.

## Background

A patient should not expect to receive a lower standard of care because of her race, age or any other irrelevant characteristic. However, implicit associations (unconscious, uncontrollable, or arational processes) may influence our judgements resulting in bias. Implicit biases occur between a group or category attribute, such as being black, and a negative evaluation (implicit prejudice) or another category attribute, such as being violent (implicit stereotype) [1].^1^ In addition to affecting judgements, implicit biases manifest in our non-verbal behaviour towards others, such as frequency of eye contact and physical proximity. Implicit biases explain a potential dissociation between what a person explicitly believes and wants to do (e.g. treat everyone equally) and the hidden influence of negative implicit associations on her thoughts and action (e.g. perceiving a black patient as less competent and thus deciding not to prescribe the patient a medication).

The term ‘bias’ is typically used to refer to both implicit stereotypes and prejudices and raises serious concerns in healthcare. Psychologists often define bias broadly; such as ‘the negative evaluation of one group and its members relative to another’ [2]. Another way to define bias is to stipulate that an implicit association represents a bias only when likely to have a negative impact on an already disadvantaged group; e.g. if someone associates young girls with dolls, this would count as a bias. It is not itself a negative evaluation, but it supports an image of femininity that may prevent girls from excelling in areas traditionally considered ‘masculine’ such as mathematics [3]. Another option is to stipulate that biases are not inherently bad, but only to be avoided when they incline us away from the truth [4].

In healthcare, we need to think carefully about exactly what is meant by bias. To fulfil the goal of delivering impartial care, healthcare professionals should be wary of any kind of negative evaluation they make that is linked to membership of a group or to a particular characteristic. The psychologists’ definition of bias thus may be adequate for the case of implicit prejudice; there are unlikely, in the context of healthcare, to be any justified reasons for negative evaluations related to group membership. The case of implicit stereotypes differs slightly because stereotypes can be damaging even when they are not negative per se. At least at a theoretical level, there is a difference between an implicit stereotype that leads to a distorted judgement and a legitimate association that correctly tracks real world statistical information. Here, the other definitions of bias presented above may prove more useful.

The majority of people tested from all over the world and within a wide range of demographics show responses to the most widely used test of implicit attitudes, the Implicit Association Test (IAT), that indicate a level of implicit anti-black bias [5]. Other biases tested include gender, ethnicity, nationality and sexual orientation; there is evidence that these implicit attitudes are widespread among the population worldwide and influence behaviour outside the laboratory [6, 7]. For instance, one widely cited study found that simply changing names from white-sounding ones to black-sounding ones on CVs in the US had a negative effect on callbacks [8]. Implicit bias was suspected to be the culprit, and a replication of the study in Sweden, using Arab-sounding names instead of Swedish-sounding names, did in fact find a correlation between the HR professionals who preferred the CVs with Swedish-sounding names and a higher level of implicit bias towards Arabs [9].

We may consciously reject negative images and ideas associated with disadvantaged groups (and may belong to these groups ourselves), but we have all been immersed in cultures where these groups are constantly depicted in stereotyped and pejorative ways. Hence the description of ‘aversive racists’: those who explicitly reject racist ideas, but who are found to have implicit race bias when they take a race IAT [10]. Although there is currently a lack of understanding of the exact mechanism by which cultural immersion translates into implicit stereotypes and prejudices, the widespread presence of these biases in egalitarian-minded individuals suggests that culture has more influence than many previously thought.

The implicit biases of concern to health care professionals are those that operate to the disadvantage of those who are already vulnerable. Examples include minority ethnic populations, immigrants, the poor, low health-literacy individuals, sexual minorities, children, women, the elderly, the mentally ill, the overweight and the disabled, but anyone may be rendered vulnerable given a certain context [11]. The vulnerable in health-care are typically members of groups who are already disadvantaged on many levels. Work in political philosophy, such as the De-Shalit and Wolff concept of ‘corrosive disadvantage’, a disadvantage that is likely to lead to further disadvantages, is relevant here [12]. For instance, if a person is poor and constantly worried about making ends meet, this is a disadvantage in itself, but can be corrosive when it leads to further disadvantages. In a country such as Switzerland, where private health insurance is mandatory and yearly premiums can be lowered by increasing the deductible, a high deductible may lead such a person to refrain from visiting a physician because of the potential cost incurred. This, in turn, could mean that the diagnosis of a serious illness is delayed leading to poorer health. In this case, being poor is a corrosive disadvantage because it leads to a further disadvantage of poor health.

The presence of implicit biases among healthcare professionals and the effect on quality of clinical care is a cause for concern [13–15]. In the US, racial healthcare disparities are widely documented and implicit race bias is one possible cause. Two excellent literature reviews on the issue of implicit bias in healthcare have recently been published [16, 17]. One is a narrative review that selects the most significant recent studies to provide a helpful overall picture of the current state of the research in healthcare on implicit bias [16]. The other is a systematic review that focusses solely on racial bias and thus captures only studies conducted in the US, where race is the most prominent issue [17]. Our review differs from the first because it poses a specific question, is systematic in its collection of studies, and includes an examination of studies solely employing the vignette method. Its systematic method lends weight to the evidence it provides and its inclusion of the vignette method enables it to compare two different literatures on bias in healthcare. It differs from the second because it includes all types of bias, not only racial; partly as a consequence, it captures many studies conducted outside the US. It is important to include studies conducted in non-US countries because race understood as white/black is not the source of the most potentially harmful stereotypes and disparities in all cultural contexts. For example, a recent vignette study in Switzerland found that in the German-speaking part of the country, physicians displayed negative bias in treatment decisions towards fictional Serbian patients (skin colour was unspecified, but it would typically be assumed to be white), but no significant negative bias towards fictional patients from Ghana (skin colour would be assumed to be black) [18]. In the Swiss German context, the issue of skin colour may thus be less significant for potential bias than that of country of origin.^2^

## Methods

### Data sources and search strategy

Our research question was: do trained healthcare professionals display implicit biases towards certain types of patient? PubMed (Medline), PsychINFO, PsychARTICLE and CINAHL were searched for peer-reviewed articles published between 1st March 2003 and 31st March 2013. When we performed exploratory searches on PubMed before conducting the final search, we noticed that in 2003 there was a sharp increase in the number of articles on implicit bias and so we decided to begin from this year. The final searches were conducted on the 31st March 2013. We used a combination of subject headings and free text terms that related to the attitudes of healthcare professionals (e.g. “physician-patient relations”, “attitude of health personnel”), implicit biases (e.g. “prejudice”, “stereotyping”, “unconscious bias”), particular kinds of discrimination (e.g. “aversive racism”, anti-fat bias”, “women’s health”), and healthcare disparities (e.g. “health status disparities”, “delivery of health care”) which were combined with the Boolean operators “AND” and “OR”.

### Study selection

3767 titles were retrieved and independently screened by the two reviewers (SH and CF). The titles that were agreed by both after discussion to be ineligible according to our inclusion criteria were discarded (3498) and the abstracts of the remaining articles (269) were independently screened by both reviewers. Abstracts that were agreed by both reviewers to be ineligible according to our inclusion criteria were discarded (241). When the ineligible abstracts were discarded, the remaining 28 articles were read and independently rated by us both. Out of these, 27 articles were agreed after discussion to merit inclusion in the final selection. One article was excluded at this stage because it did not fit our inclusion criteria (it did not employ the assumption method or an implicit measure). Additionally, the reference lists of these 27 articles were manually scanned by CF, and the full text articles resulting from this were independently read by both reviewers, resulting in the inclusion of a further 11 articles that both reviewers agreed fitted the inclusion criteria. After a repeat process of scanning the reference lists of the 11 articles from the second round, the final number of eligible articles was 42. All disagreements were resolved through discussion.

The inclusion criteria were:

1. Empirical study.
2. A method identifying *implicit* rather than *explicit* biases.
3. Participants were physicians or nurses who had completed their studies.
4. Written in English or another language spoken by CF or SH (CF: French, Italian, Spanish, Catalan; SH: French, Italian, German).

There is no clear consensus on the meaning of the term ‘implicit’. The term is used in psychology to refer to a feature or features of a mental process. We chose a wide negative definition of implicit processes, assuming that implicit social cognition is involved in the absence of any of the four features that characterise explicit cognition: intention, conscious availability, controllability, and the need for mental resources. This absence does not rule out the involvement of explicit processes, but indicates the presence of implicit processes. While most institutional policies against bias focus on explicit cognition, research on implicit bias shows that this is mistaken [6].

There is broad agreement in psychology that methods known as ‘implicit measures’, including the affective priming task, the IAT and the affective Simon task, reveal implicit attitudes [19]. We included articles using these measures. We also included studies that employed a method popular in bioethics literature that we label ‘the assumption method’. It involves measuring differences across participants in response to clinical vignettes, identical except for one feature, such as the race, of the character in the vignette. There is no direct measure of the implicitness or non-explicitness of the processes at work in participants; instead, there is an assumption that the majority are explicitly motivated to disregard factors such as race. If there is a statistically significant difference in the diagnosis or treatment prescribed correlated with –for example- the race of the patient, the researchers infer that it is partly a result of implicit processes in the physicians’ decision-making. The assumption method of measuring implicit bias has been used in a variety of naturalistic contexts where it is harder to bring subjects into the laboratory. It is recognised by psychologists who specialise in implicit cognition as a way of detecting the possible presence of implicit bias, if not as an implicit measure in itself [6].

Studies that used self-report questionnaires were not included because, although they can use subtle methods to estimate a subject’s attitudes, they are typically used in psychology as a measure of explicit mental processes. There are potential problems with the implicit/explicit distinction as applied to psychological measures and it may be preferable in future research to speak of ‘direct’ and ‘indirect’ measures, but for the purposes of the review we followed this convention in psychology. The original idea behind implicit measures was that they attempted to measure something other than explicit mental processes, whereas self-report questionnaires ask a subject direct questions and thus prompt a chain of explicit conscious reasoning in the subject.

### Data extraction

Data were extracted by CF and reviewed by SH for accuracy and completeness. All disagreements with the information extracted were resolved through discussion. We contacted the corresponding author of an article to obtain information that was not available in the published manuscript that related to the nature of the presentation given to recruit participants, but received no response.

## Results

### Identified studies

The eligible studies are described in Table 1 and their main characteristics are outlined in Table 2. The most frequently examined biases were racial/ethnic and gender, but ten other biases were investigated (Table 2). Four of the assumption studies compared results from two or more countries to explore effects of differences in healthcare systems.

**Table 1:** Studies included in the systematic review

Year	First author	Country	Method	Population	Recruitment	Response rate	Main findings (relevant to systematic review)
Age							
2010	Protière [25]	France	Assumption Method	388 oncologists and radiotherapists	Mail-out countrywide.	69%	Significant negative differences in treatment choice for older patients.
AIDS patients							
2007	Li [61]	China	Assumption Method	1101 (including just over 50% doctors, 40% nurses)	Random selection of institutions and individuals.	Less than 8% refusal rate (includes 10% lab technicians)	Attitudes as measured across subjects were more negative towards AIDS patients than towards hepatitis B patients.
Brain injured patients who have contributed to their injury							
2011	Linden [58]	UK	Assumption Method	69 nurses	Email.	24%	More negative attitudes as measured across subjects were found against individuals seen as contributing towards their injury.
2010	Redpath [59]	UK	Assumption Method	155 (94 qualified nurses, 61 qualified doctors)	Not specified.	Not specified.	More negative attitudes as measured across subjects were found against individuals seen as contributing towards their injury. Attitudes significantly related to intended helping behaviour.
Disability							
2012	Aaberg [62]	US	IAT (prejudice)	132 nurse educators	Email.	21%	Negative implicit bias against the disabled, stronger than that of the average population.
Gender							
2005	Abuful [63]	Israel	Assumption Method	172 physicians (internists, cardiologists, family physicians, general practitioners)	Continuing medical education.	Not specified.	Negative bias against women in diagnosis of risk and prescription of lipid-lowering medications and aspirin.
Intravenous Drug Users (IDUs)							
2007	Brener [49]	Australia	IAT (prejudice)	60 health care workers (HCW) from drug and alcohol facilities and liver clinics: 21 physicians, 37 nurses, two medical students	Different facilities and GPs identified through networking.	Not specified.	HCW had positive explicit attitudes and negative implicit attitudes towards HCV positive IDUs. Contact (as estimated by HCW) predicted explicit (positive) and implicit attitudes (negative) towards IDU beyond the effect of conservatism.
2008	von Hippel [48]	Australia	IAT (prejudice)	44 Drug and Alcohol (D&A) nurses	Selection of Drug & Alcohol treatment facilities, needle and syringe exchange programs, and primary-care facilities.	Not specified.	Challenging behaviours by IDUs predicted self-reported stress of nurses, which, in turn, predicted intention to change jobs. The relation between stress and intention to change jobs significantly mediated by the nurses’ implicit prejudice, not explicit prejudice.
Mentally ill							
2007	Chow [64]	Hong Kong	Assumption Method	433 (107 physicians, 322 nurses and four who didn't state)	Random distribution via ward managers (nurses) and email (physicians).	36.1%	More negative attitudes as measured across subjects found towards psychiatric patients than to non-psychiatric patients.
2005	Mackay [26]	UK	Assumption Method	89 A&E medical and nursing staff	4 A&E Departments in Greater Manchester.	49%	The greater the attributions of controllability to self-harming patients, the greater the negative affect of staff towards the patient as measured across subjects, and the less the propensity to help.
2012	Neauport [65]	France	Assumption Method	322 medical residents of all specialties in one hospital	Email.	47.4%	Those assigned the vignette that included the psychiatric illness label said that they were less likely to want to treat the individual and be involved with her/him in various ways.
2008	Peris [42]	Worldwide	IAT (prejudice and stereotype)	682 mental health professionals (clinical psychologists, social workers, counsellors, psychiatrists and others) and clinical graduate students.	Project Implicit website and 110 clinicians and graduate students recruited directly through list serves.	81% (after random assignment to study via Project Implicit and including 747 non-mental health professionals)	Overall, explicit and implicit views were not negative towards individuals with mental illness. Those with mental health training displayed less implicit and explicit prejudices. Their explicit (but not implicit) biases predicted more negative patient prognoses, but implicit (and not explicit) biases predicted over-diagnosis.
Multiple biases							
2004	Arber^a^ [27]	US/UK	Assumption Method Biases: Age, Gender, Racial/ethnic and Socio-Economic Status (SES)	256 primary care physicians in the US and the UK	Screening telephone calls.	65% in the US and 60% in the UK.	Gender and age influenced the doctors’ questioning of patients presenting with coronary heart disease (CHD) in both countries. Men were asked more questions overall and middle-aged patients were asked more lifestyle questions.
2006	Arber^a^ [32]	UK and US	Assumption Method Biases: Age, Gender, Racial/ethnic and SES.	256 primary care physicians in the US and the UK	Screening telephone calls.	65% in the US and 60% in the UK.	The gender of the patient significantly influenced doctors’ diagnostic and management activities. Midlife women were asked fewest questions and prescribed least medication appropriate for CHD.
2006	Barnhart [66]	US	Assumption Method Biases: Racial/ethnic, Gender, and Social Circumstances.	544 family medicine physicians, internists, cardiologists, and cardiothoracic surgeons	Mail-out.	70%	The patient’s race and gender did not significantly affect the physicians’ treatment preferences. However, significant differences were found according to social circumstance.
2008	Bönte^a^ [31]	US, UK and Germany	Assumption Method Biases: Age, Gender, Racial/ethnic and SES.	384 physicians (internists or family practitioners in the US and Germany or general practitioners in the UK)	Screening telephone calls.	64.9% in the US, 59.6% in the UK, and 65% in Germany.	Results showed gender differences in the diagnostic strategies of the doctors.
2010	Dehlendorf [39]	US	Assumption Method Biases: Racial/ethnic and SES.	524 health care providers (96% MD/DO, 4% Nurse Practitioner/Physician Assistant)	Convenience sample from meetings of professional societies.	Not specified.	Low SES whites were less likely to have intrauterine contraception recommended than high SES whites. By race/ethnicity, low SES Latinas and blacks were more likely to have intrauterine contraception recommended than low SES whites, with no effect of race/ethnicity for high SES patients. Low SES patients were judged to be significantly more likely than high SES patients to have an STI and an unintended pregnancy, and were also judged to be less knowledgeable.
2005a	Kales [37]	US	Assumption Method Biases: Racial/ethnic and Gender.	321 psychiatrists	Attendees at the 2002 annual meeting of the American Psychiatric Association.	Not specified.	Patients’ race and gender was not associated with significant differences in the diagnoses of major depression. However, white patients were rated as being of significantly higher SES than black patients. A significant relationship was found between rating of SES and estimates of patient demeanour (lower SES associated with more hostile demeanour).
2005b	Kales [38]	US	Assumption Method Biases: Racial/ethnic and Gender.	178 Primary Care Physicians (PCPs)	Attendees at the 2002 American Academy of Family Physicians Annual Meeting.	Not specified.	Patients’ race and gender was not associated with significant differences in the diagnoses of major depression. However, white patients were rated as being of significantly higher SES than black patients.
2009a	Lutfey^a^ [29]	US, UK and Germany	Assumption Method Biases: Age, Gender, Racial/ethnic and SES.	384 physicians (internists or family practitioners in the US and Germany and general practitioners in the UK)	Screening telephone calls.	64.9% in the US, 59.6% in the UK, and 65.0% in Germany	Physicians were least certain of CHD diagnoses when patients were younger and female. Certainty was positively correlated with several clinical actions, including test ordering, prescriptions, referrals to specialists, and time to follow-up.
2009b	Lutfey^a^ [35]	US	Assumption Method Biases: Age, Gender, Racial/ethnic and SES	128 generalist physicians	Screening telephone calls.	64.9%	Physicians were least certain of their CHD diagnoses for black patients and for younger women. Physicians responded differentially to diagnostic certainty in terms of their clinical therapeutic actions such as test ordering and writing prescriptions.
2010	Lutfey [33]	US	Assumption Method Biases: Age, Gender, Racial/ethnic and SES.	256 internists or family/general practitioners	Screening telephone calls.	Not specified.	Physicians primed with CHD were more likely to order CHD-related tests and prescriptions. Main effects for patient gender and age remained, suggesting that physicians treated these demographic variables as diagnostic features indicating lower risk of CHD for these patients.
2009a	Maserejian^a^ [34]	US	Assumption Method Biases: Age, Gender, Racial/ethnic and SES.	128 physicians (internist or family practitioner)	Screening telephone calls.	Not specified.	Physicians were significantly less certain of the underlying cause of symptoms among female patients regardless of age, but only among middle-aged women were they significantly less certain of the CHD diagnosis.
2009b	Maserejian [36]	US	Assumption Method Biases: Age, Gender, Racial/ethnic and SES.	256 internists or family/general practitioners	Screening telephone calls.	Not specified.	48% of physicians were inconsistent in their population-level and individual-level CHD assessments. Physicians’ assessments of CHD prevalence did not attenuate the observed gender effect in diagnostic certainty for the individual patient.
2006	McKinlay^a^ [28]	US/UK	Assumption Method Biases: Age, Gender, Race and SES.	256 primary care physicians in the US and the UK	Screening telephone calls.	64.9% in the US and 59.6% in the UK.	Age, race, gender, but not SES, influenced decision-making for the conditions studied in both countries. Differences were also found between treatment decisions in the UK and the US.
2007	McKinlay^a^ [30]	US	Assumption Method Biases: Age, Gender, Racial/ethnic and SES.	128 internists and family physicians	Screening telephone calls.	64.9%	Female patients were less likely than males to receive 4 of 5 types of physical examination; older patients were less likely to be advised to stop smoking. Race and SES of patients had no effect on provider adherence to guidelines.
2003	Tamayo-Sarver [40]	US	Assumption Method Biases: Racial/ethnic	2872 emergency physicians	Mail-out.	53%	The race/ethnicity of patients in the vignettes had no effect on physician prescription of opioids. Making socially desirable information explicit increased the prescribing rates by 4% for the migraine vignette and 6% for the back pain vignette.
Racial/ethnic							
2011	Barnato [41]	US	Assumption Method	33 hospital-based physicians (emergency physicians, hospitalists, intensivists)	Probability sampling (15) and convenience sampling (18).	Reported as ‘low’.	Physicians did not make different treatment decisions for black and white patients, despite believing that black patients were more likely to prefer intensive, life-sustaining treatment.
2013a	Blair [44]	US	IAT (prejudice) and interpersonal interaction measures	134 clinicians	Data for clinicians collected in the Blair [44] study. Primary data from patients in a broader study on hypertension.	60%	Clinicians with greater implicit bias against blacks were rated lower in patient-centred care by their black patients as compared with a reference group of white patients.
2013b	Blair [22]	US	IAT (prejudice)	210 experienced primary care providers (PCPs)	Invitation launched with presentations.	60%	Substantial implicit bias against Latinos and African Americans in PCPs
2008	Burgess [43]	US	Assumption Method	382 general internal medicine physicians	Mail-out.	40%	There was no significant effect of patient race alone. Among black patients, physicians were significantly more likely to state that they would switch to a higher dose or stronger opioid for patients exhibiting “challenging” behaviours compared to those exhibiting “non-challenging” behaviours.
2012	Cooper [23]	US	IAT (prejudice and stereotype), audiotape measures of visit communication and patient ratings.	40 primary care clinicians (36 physicians, four nurses) in urban community-based practices.	Secondary data from two previous studies, where patients and providers participated in randomised clinical trials of interventions to enhance communication.	63%	Clinician implicit race bias and race and compliance stereotyping were associated with markers of poor visit communication and poor ratings of care, particularly among black patients.
2007	Green [46]	US	IAT (prejudice and stereotype) and vignettes	287 physicians	Email.	50.6%	All 3 IATs showed significant race bias. Physicians were more likely to diagnose black patients than white patients with CAD. However, physicians were equally likely to give thrombolysis for black and white patients. There was thus a racial disparity in thrombolysis relative to CAD diagnosis.
2012	Moskowitz [20]	US	Subliminal priming (faces).	Study 1: 16 physicians. Study 2: 11 physicians	Convenience sample.	Not specified.	When primed with a black face, physicians reacted more quickly for stereotypical diseases, indicating an implicit association of certain diseases with black patients. These comprised not only diseases that black patients are genetically predisposed to, but also conditions and social behaviours with no biological association (e.g. obesity, drug use).
2010	Penner [24]	US	IAT (prejudice) and interaction measures	15 resident physicians (3 white and 12 self-identified as Indian, Pakistani or Asian)	Patients recruited consecutively and physicians invited.	83% physicians	Overall, physicians did not display implicit race bias. However, black patients had less positive reactions to medical interactions with physicians relatively low in explicit but relatively high in implicit bias than to interactions with physicians who were either (a) low in both explicit and implicit bias, or (b) high in both explicit and implicit bias.
2008	Sabin^b^ [47]	US	IAT (prejudice and stereotype) and vignettes	86 academic paediatricians from one department	Invited all faculty, residents and fellows at a large, urban research university to participate.	58%	Paediatricians held implicit race bias, but it was weaker than that of other MDs and others in society.
2009	Sabin [67]	Worldwide	IAT (prejudice)	2535 MDs	Project Implicit website.	Not applicable	Medical doctors, like the rest of the sample, showed a strong implicit preference for whites over blacks.
2012a	Sabin^b^ [45]	US	IAT (prejudice and stereotype) and vignettes	86 academic paediatricians from one department.	Invited all faculty, residents and fellows at a large, urban research university to participate.	58%	Paediatricians’ implicit bias was associated with treatment recommendations. As paediatricians’ implicit pro-white bias increased, prescribing narcotic medication decreased for black patients, but not for white patients.
2012	Stepanikova [21]	US	Subliminal priming (words) and vignettes	81 family physicians and general internists	Email.	2%	Under higher time pressure, but not lower, implicit biases against blacks and Hispanics led to less serious diagnosis. Under higher time pressure, implicit bias against blacks led to lower rate of referral to specialist.
Weight							
2012b	Sabin [60]	Worldwide	IAT (prejudice)	2284 medical doctors (MDs)	Project Implicit website.	Not applicable.	MDs, like the wider population tested, had a strong implicit anti-fat bias and a strong explicit anti-fat bias.
2003	Schwartz [50]	Canada	IAT (prejudice and stereotype)	389 (122 physicians, 12 psychologists, five nurses, 18 other obesity clinicians)	Attendees of the Annual Meeting of the North American Association for the Study of Obesity.	Not specified.	There was a significant implicit anti-fat prejudice and stereotype found.
2007	Vallis [68]	Canada	IAT (prejudice and stereotype)	78 (14.3% physicians, 15.4% nurses)	Attendees of on obesity conference.	86% of total attendees.	Strong evidence for anti-fat prejudice and stereotype.

^a^What appear to be the same data from the US, the UK and Germany are selectively analysed in different ways in these eight studies

^b^What appear to be the same data are analysed in different ways in these two studies

**Table 2:** Main characteristics of studies

	Studies (*N* = 42)	
Method		
Assumption method	25 [25–41, 43, 58, 59, 61, 63–66]	
Implicit measure	17 [20–24, 42, 44–50, 60, 62, 67, 68]	
Implicit measure - IAT	15 [22–24, 42, 44–50, 60, 62, 67, 68]	
	(of which: IAT combined with behaviour or decision)	9 [23, 24, 42, 44–48, 67]
Implicit measure - Subliminal priming	2 [20, 21]	
Setting and type of test		
IAT – implicit prejudice	15 [22–24, 42, 44–50, 60, 62, 67, 68]	
IAT – implicit stereotype	5 [23, 45–47, 50]	
IAT – standard	13 [22–24, 42, 44–47, 50, 60, 62, 67, 68]	
IAT – Single Category	2 [48, 49]	
IAT – uncontrolled setting	10 [22, 23, 42, 45–47, 50, 60, 62, 67]	
IAT - controlled laboratory setting	3 [48, 49, 68]	
IAT – setting unspecified	2 [24, 44]	
Assumption method – video vignette with oral questions	10 [27–36]	
Assumption method – written texts	11 [25, 26, 40, 43, 58, 59, 61, 63–66]	
	(of which: photos in addition)	1 [43]
Assumption method – video vignette with written questions	3 [37–39]	
Assumption method – simulations of encounters with patients and role-play	1 [41]	
Assumption method – controlled setting	16 [27–39, 41, 58, 63]	
Assumption method – uncontrolled setting	8 [25, 26, 40, 43, 61, 64–66]	
Assumption method – setting unspecified	1 [59]	
Bias(es) studied		
Racial/ethnic	27 [20–24, 27–41, 43–47, 67]	
Multiple	14 [27–39, 66]	
Gender	14 [27–38, 63, 66]	
Socio-economic status (SES)	11 [27–36, 39]	
Age	11 [25, 27–36]	
Mental illness	4 [26, 42, 64, 65]	
Weight	3 [50, 60, 68]	
Brain-injured patients perceived to have contributed to their injury	2 [58, 59]	
Intravenous drug users	2 [48, 49]	
Disability	1 [62]	
AIDS patients	1 [61]	
Social circumstances (desiring an active lifestyle, having a demanding career, having family demands)	1 [66]	
Country of study		
US	27 [20–24, 27–41, 43–47, 62, 66]	
UK	8 [26–29, 31, 32, 58, 59]	
Compared countries (US, UK and Germany)	4 [27–29, 31]	
Worldwide	3 [42, 60, 67]	
France	2 [25, 65]	
Australia	2 [48, 49]	
Germany	2 [29, 31]	
Canada	2 [50, 68]	
Israel	1 [63]	
Hong Kong	1 [64]	
China	1 [61]	
	Participants (*N* = 15148)	
Profession of participants		
Physicians	12156	
Nurses	740	
Either physicians or nurses	1404	
‘Clinicians’, or ‘mental health professionals’ (at least some of whom were nurses and physicians)	834	
Psychologists	12	
Medical Students	2	

The 14 assumption method studies examining multiple biases investigated interactions between biases. They recorded the socio-demographic characteristics of the participants to reveal complex interactions between physician characteristics and the characteristics of the imaginary ‘patient’ in the vignette.

All IAT studies measured implicit prejudice; five also measured implicit stereotypes. When implicit prejudice is measured, words or images from one category are matched with positive or negative words (e.g., black faces with ‘pleasant’). When implicit stereotypes are measured, words or images from one category are matched with words from a conceptual category (e.g. female faces and ‘home’).

Nine IAT studies combined the IAT with a measure of physician behaviour or treatment decision to see if there were correlations between these and levels of implicit bias.

The subliminal priming studies were dissimilar: one was an exploratory study to see if certain diseases were stereotypically associated with African Americans, using faces as primes and reaction times to the names of diseases as the measure of implicit association; the other study used race words as primes and tested the effect of time pressure on responses to a clinical vignette.

A variety of media were used for the clinical vignette and the method of questioning participants within the assumption method. One unusual study used simulations of actual encounters with patients, hiring actors and using a set for the physicians to role-play. Physicians’ treatment decisions were recorded by observers, and the physician recorded his own diagnosis, prognosis and perceptions after the encounter.

### Limitations

#### Of specific studies

Limitations are detailed in Table 3. Some studies failed to report response rates, or to provide full information on statistical methods or participant characteristics. Some had very small sample sizes and the majority did not mention calculating the power of their sample. Some authors explicitly informed participants of the purpose of the study, or gave participants questionnaires or other tests that indicated the subject of the study before presenting them with the vignette. For optimal results, participants should not be alerted to the particular patient characteristic(s) under study, particularly in an assumption study where knowing the characteristic(s) may influence the interpretation of the vignette. In IAT studies, this is less worrying because IAT effects are to some extent uncontrollable.

**Table 3:** Limitations of specific studies

Recruitment method not reported	1 [59]
Failed to report response rate	12 [20, 33, 34, 36–39, 48–50, 59, 63]
Response rate reported as ‘low’	1 [41]
Response rate less than 40%	7 [21, 26, 43, 58, 62, 64, 65]
Explicitly informed participants of the purpose of the study	7 [25, 27, 32, 42, 58, 60, 67]
Gave participants tests or questionnaires that indicated patient characteristic under scrutiny prior to vignette	2 [48, 49]
Did not specify whether they informed participants about the purpose of the study	16 [22, 24, 28–31, 34–36, 39, 44, 59, 61, 62, 64, 66]
Small sample size	3 [20, 21, 48]
Failed to report calculating power when designing study	All studies failed except the 15 referenced here that did [27–36, 39, 40, 42, 43, 59]
Full information on statistical methods used not provided	4 [32, 49, 61, 63]

#### Of the field

Implicit bias in healthcare is an emerging field of research with no established methodology. This is to be expected and is not a problem in itself, but it does present an obstacle when conducting a review of this kind. The range of methods used and the variety of journals with differing standards and protocols for describing experiments made it difficult to compare the results. In addition, authors focusing on a particular bias (e.g. gender), often in combination with a particular health issue (e.g. heart disease), frequently did not appear to be familiar with one another’s research. This lack of familiarity meant that often used different terms to describe the same phenomenon, which also made conducting the review more difficult.

Few of the existing results can be described as ‘real world’ treatment outcomes. The two priming studies involved very small samples and were more exploratory than result-seeking [20, 21]. The IAT and assumption studies were conducted under laboratory conditions. The only three studies conducted in naturalistic settings combined the IAT with measures of physician-patient interaction [22–24]. However, many of the assumption studies attempted to make their vignettes as realistic as possible by having them validated by clinicians [25–41] and also by having participants view/read the vignettes as part of a normal day at work [27–36, 39, 41].

Because the studies of interest used psychological techniques, but were mainly to be found in a medical database (PubMed), the classification of the studies was not always optimal. There is no heading in Medline for ‘implicit bias’ and studies using similar methods were sometimes categorized under different subject headings, some of which were introduced during the last ten years, which increased the risk of missing eligible studies.

### Existence of implicit biases/stereotypes in healthcare professionals and influence on quality of care

#### Healthcare professionals have implicit biases

Almost all studies found evidence for implicit biases among physicians and nurses. Based on the available evidence, physicians and nurses manifest implicit biases to a similar degree as the general population. The following characteristics are at issue: race/ethnicity, gender, socio-economic status (SES), age, mental illness, weight, having AIDS, brain injured patients perceived to have contributed to their injury,^3^ intravenous drug users, disability, and social circumstances.

Of the seven studies that did not find evidence of bias, one compared the mentally ill with another potentially unfavourable category, welfare recipients; this study did find a positive correlation between levels of implicit bias and over-diagnosis of the mentally ill patient in the vignette [42]. Another used simulated interactions with actors, which may result in participants being on ‘best behaviour’ in the role-play [41]. The two studies that reported no evidence of bias in diagnosis of depression found that physicians’ estimates of SES were influenced by race (lower SES estimated for black patients); [37, 38] one reported that estimates of SES in turn were significantly related to estimates of patient demeanour (lower SES associated with hostile patient demeanour) [37]. A further study failed to find differences due to patient race in the prescription of opioids, but found an interaction whereby black patients who exhibited ‘challenging’ behaviour (such as belligerence and asking for a specific opioid) were more likely to be prescribed opioids than those who did not, an effect possibly due to a racial stereotype [43]. Another study that failed to find implicit race bias suggested that this was due to the setting of the study in an inner-city clinic with high levels of black patients and the fact that many physicians were born outside the US [24]. Finally, one study that found no evidence of racial bias in prescription of opioid analgesics presented each participant with three vignettes depicting patients of three different ethnicities, thus probably alerting them to the objective of the study [40].

The interaction effects between different patient characteristics in assumption studies are varied and a few are surprising. The authors of one study expected that physicians would be less likely to prescribe a higher dose of opioids to black patients who exhibited challenging behaviours; in fact, physicians were more likely to prescribe higher doses of opioids to challenging black patients, yet slightly less likely to do so to white patients exhibiting the same behaviour. Sometimes significant effects on the responses to the vignette of a patient characteristic, e.g. race, are only found when the interaction between gender and race or SES and race is examined. For example, physicians in one study were less certain of the diagnosis of coronary heart disease for middle-aged women, who were thus twice as likely to receive a mental health diagnosis than their male counterparts [34]. In another, low SES Latinas and blacks were more likely to have intrauterine contraception recommended than low SES whites, but there was no effect of race for high SES patients [39].

#### Implicit bias affects clinical judgement and behaviour

Three studies found a significant correlation between high levels of physicians’ implicit bias against blacks on IAT scores and interaction that was negatively rated by black patients [23, 24, 44] and, in one study, also negatively rated by external observers [23]. Four studies examining the correlation between IAT scores and responses to clinical vignettes found a significant correlation between high levels of pro-white implicit bias and treatment responses that favoured patients specified as white [42, 45–47]. In one study, implicit prejudice of nurses towards injecting drug users significantly mediated the relationship between job stress and their intention to change jobs [48].

Twenty out of 25 assumption studies found that some kind of bias was evident either in the diagnosis, the treatment recommendations, the number of questions asked of the patient, the number of tests ordered, or other responses indicating bias against the characteristic of the patient under examination.

#### Determinants of bias

Socio-demographic characteristics of physicians and nurses (e.g. gender, race, type of healthcare setting, years of experience, country where medical training received) are correlated with level of bias. In one study, male staff were significantly less sympathetic and more frustrated than female staff with self-harming patients presenting in A&E [26]. Black patients in the US –but not the UK- were significantly more likely to be questioned about smoking than white [28]. In another study, international medical graduates rated the African-American male patient in the vignette as being of significantly lower SES than did US graduates [38]. One study found that paediatricians held less implicit race bias compared with other MDs [47].

Correlations between explicit and implicit attitudes varied depending on the type of bias and on the kind of explicit questions asked. For instance, implicit anti-fat bias tends to correlate more with an explicit anti-fat bias than racial bias, where explicit and implicit attitudes often diverge significantly. Because physicians’ and nurses’ implicit attitudes diverged frequently from their explicit attitudes, explicit measures cannot be used alone to measure the presence of bias among healthcare professionals.

## Discussion

A variety of studies, conducted in various countries, using different methods, and testing different patient characteristics, found evidence of implicit biases among healthcare professionals and a negative correlation exists between level of implicit bias and indicators of quality of care. The two most common methods employed were the assumption method and the IAT, the latter sometimes combined with another measure to test for correlations with the behaviour of healthcare professionals.

Our study has several limitations. Four studies included participants who were not trained physicians or nurses and failed to report separate results for these categories of participants [42, 44, 49, 50]. Since either the majority of participants were qualified physicians and nurses, or were other health care professionals involved in patient care, we included these studies despite this limitation. Excluding them would not have changed the conclusions of this paper. In addition, we initially centred our research on studies employing implicit measures recognised in psychology, but the majority of the included studies in the final review used the assumption method. However, the limitations imposed by the lack of consistency in keywords and categorization of articles actually worked in our favour here, enabling us to capture a variety of methods and thus to consider including the assumption method. Scanning the references of the articles that were initially retained and repeating this process until there were no new articles helped us to capture further pertinent articles. From the degree of cross-referencing we are confident that we succeeded in identifying most of the relevant articles using the assumption method.

Publication bias could limit the availability of results that reveal little or no implicit bias among healthcare professionals. Moreover, eight articles appeared to refer to the same data collected in a single cross-country comparison study [27–32, 34, 35] and a further two articles analysed the same data [45, 47]. The sum of 42 articles thus can give the impression that more research has been carried out on more participants than is the case. The solidity of data revealing high levels of implicit bias among the general population suggest that this is unlikely to have invalidated the conclusion that implicit bias is present in healthcare professionals [6, 7].

However, our decision to exclude studies that involved students rather than fully-trained healthcare professionals meant that we did not include a study conducted on medical students that showed no significant association between implicit bias and clinical assessments [51]. Several studies post 2013 (thus after our cut-off date) have also indicated a null relationship between levels of implicit bias and clinical decision-making [52–54]. The scientific community working in this area agrees that the relationship between levels of implicit bias in healthcare professionals and clinical decision-making is complex and that there is currently a lack of good evidence for a direct negative influence of biases [16, 17]. As our review shows, there is clearer evidence for a relationship between implicit bias and negative effects on clinical interaction [23, 24, 44]. While this may not always translate into negative treatment outcomes, the relationship between a healthcare professional and her patient is essential to providing good treatment, thus it seems likely that the more negative the clinical interaction, the worse the eventual treatment outcome (not to mention the likelihood that the patient will consult healthcare services for future worries or problems). This is where the bulk of future research should be concentrated.

The interactions between multiple patient characteristics and between healthcare professional and patient characteristics reveal the complexity of the phenomenon of implicit bias and its influence on clinician-patient interaction. They also highlight the pertinence of work in feminist theory on ‘intersectionality’, a term for the distinctive issues that arise when a person belongs to multiple identity categories that bring disadvantage, such as being both black and female [55]. For instance, one study only found evidence of bias against low SES Latina patients, not against high SES Latinas, illustrating how belonging to more than one category (here, both low SES and Latina) can have negative effects that are not present if membership of one category is eliminated (here, low SES) [39]. Class may trump race in some circumstances so that being high SES is more salient than being non-white. One criticism of mainstream feminism by theorists who work on intersectionality is that pertinent issues are unexplored because of the dominance of high SES white women in feminist theory. Using our example from the review, high SES Latina women may not experience the same prejudice as low SES Latina women and thus may falsely assume that there is no prejudice against Latina women *tout court* in this context. This could be frustrating for low SES Latina women who have unrecognized lived experiences of prejudice in a clinical setting.

In some studies, the attitudes of patients towards healthcare professionals were recorded and used to evaluate clinical interaction [23, 24, 44]. It is important to remember that patients also may come to a clinical interaction with biases. In these cases, the biases of one participant may trigger the biases of the other, magnifying the first participant’s biased responses and leading to a snowball effects [56]. Past experience of discrimination may mean that a patient may come to an interaction with negative expectations [57].

Our findings in the review suggest that the relationship between training and experience and levels of implicit bias is mixed. In one study, increased contact with patients with Hepatitis C virus was associated with more favourable explicit attitudes, yet more negative implicit attitudes towards intravenous drug users [49]. Another study demonstrated that nursing students were less prejudiced, more willing to help and desired more social interaction with patients with brain injury, when compared with qualified nurses [58]. Exposure to communication skills training was not associated with lower race-IAT scores for physicians [23]. However, individuals with mental health training demonstrated more positive implicit and explicit evaluations of people with mental illness than those without training [42]. Yet in the same study, graduate students had more positive implicit attitudes towards the mentally ill than mental health professionals.

We included all types of implicit bias in our review, not only race bias, partly in an effort to capture non-US studies, hypothesising that the focus on race in the US leaves fewer resources for investigation into other biases. It is possibly the case that a wider range of biases were investigated in non-US countries, but there is not enough evidence to deduce this from our review alone. For instance, two British studies examine bias against brain-injured patients who are perceived as having contributed to their injury [58, 59], and two Australian studies looked at bias against intravenous drug users [48, 49], but the sample size of studies is too small to warrant drawing any conclusions from this.

Is it possible that there are implicit associations that are justified because they are based on prevalence data for diseases? One study in our review aimed to test the statistical discrimination hypothesis by asking physicians to estimate the prevalence data among males and females for coronary heart disease in addition to presenting them with vignettes of a female or male coronary heart disease patient. It found that 48% of physicians were inconsistent in their population-level and individual level assessments and that the physicians’ gender-based population prevalence assessments were not associated with the certainty of their diagnosis of coronary heart disease. There was no evidence to support the theory of statistical discrimination as an explanation for why physicians were less certain of their diagnoses of CHD in women [36]. Another exploratory study looked at the diseases that were stereotypically associated with African-Americans and found that many diseases were associated with African-Americans that did not match prevalence data, such as drug abuse [20]. The danger in these cases is that a physician may apply a group-level stereotype to an individual and fail to follow-up with a search for individuating information.

Impartial treatment of patients by healthcare professionals is an uncontroversial norm of healthcare. Implicit biases have been identified as one possible factor in healthcare disparities and our review reveals that they are likely to have a negative impact on patients from stigmatized groups. Our review also indicates that there may sometimes be a gap between the norm of impartiality and the extent to which it is embraced by healthcare professionals for some of the tested characteristics. For instance, explicit anti-fat bias was found to be prevalent among healthcare professionals [60]. Since weight can be relevant to diagnosis and treatment, it is understandable that it is salient. It is nonetheless disturbing that healthcare professionals exhibit the same explicit anti-fat attitudes prevalent in the general population.

The most convincing studies from our review are those that combine the IAT and a method measuring the quality of treatment in the actual world. These studies provide some evidence for a relationship between bias as measured by the IAT and behaviour by clinicians that may contribute to healthcare disparities. More studies using real-world interaction measures would be helpful because studies using vignettes remain open to the criticism that they do not reveal the true behaviour of healthcare professionals. In this respect, the three studies using measures of physician-patient interaction are exemplary [22–24], in particular when using independent evaluators of the interactions [23]. Overall, our review reveals the need for discussion of methodology and for more interaction between different literatures that focus on different biases.

## Conclusion

Our findings highlight the need for the healthcare profession to address the role of implicit biases in disparities in healthcare. In addition to addressing implicit biases, measures need to be taken to raise awareness of the potential conflict between holding negative explicit attitudes towards some patient characteristics, such as obesity, and committing to a norm to treat all patients equally.

Our review reveals that this is an area in need of more uniform methods of research to enable better comparison and communication between researchers interested in different forms of bias. Important avenues for further research include examination of the interactions between patient characteristics, and between healthcare professional and patient characteristics, and of possible ways in which to tackle the presence of implicit biases in healthcare.

## Appendix 1

### Search Strategy

Pubmed

- The following combination of subject headings and free text terms was used:
  (“Prejudice” [MAJR] AND “Attitude of health personnel” [MAJR]) OR (“Attitude of health personnel/ethnology” [MH] AND “Prejudice”[MH]) OR (“Stereotyping”[MH] AND “Attitude of health personnel”) OR (“Prejudice”[MH] AND “Healthcare disparities” [MH]) OR (“Prejudice”[MH] AND “Cultural Competency” [MH]) OR (“Social Class” [MH] AND “Attitude of health personnel” [MH]) OR (“Prejudice”[MH] AND “Physicians” [MH]) OR (“Prejudice”[MAJR] AND “Delivery of Health Care”[MAJR] AND “stereotyping”[MAJR]) OR (“Physician-Patient Relations” [MH] AND “health status disparities”[MH]) OR (“Prejudice”[MH] AND “Obesity”[MH]) OR (“African Americans/psychology” [MH] AND “Healthcare disparities” [MH]) OR (“Prejudice”[MH] AND “Mentally Ill Persons”[MH]) OR (“Prejudice”[MH] AND “Women’s Health”[MH]) OR “aversive racism” OR “anti-fat bias” OR “racial-ethnic bias” OR “racial-ethnic biases” OR “ethnic/racial bias” OR “ethnic/racial biases” OR (“disabled persons”[MAJR] AND “prejudice”[MAJR])
- Dates: 1st March 2003 to 31st March 2013
- Final number of retrieved articles: 2510

PsychINFO and PsychARTICLE

- The following combination of subject headings and free text terms was used was used:
  Health personnel AND (prejudice OR bias)
- Dates: 1st March 2003 to 31st March 2013
- Other filters: Scholarly journals
- Final number of retrieved articles: 377
- Final result when duplicates removed: 360.

CINAHL

- The following combination of subject headings and free text terms was used was used:
  Prejudice [MM Exact Major Subject Heading] OR stereotyping [MM Exact Major Subject Heading] OR Discrimination [MM Exact Major Subject Heading] OR implicit bias OR unconscious bias
- Dates: 1st March 2003 to 31st March 2013
- Other filters:
  - Exclude Medline records
  - Peer reviewed
- Final number of retrieved articles: 897

## Footnotes

1 There are conceptual problems with this distinction as used in psychology that have been pointed out by philosophers, but we will ignore these for the purposes of this review.

2 Interestingly, physicians were also asked for how they expected their colleagues to rate the vignette, and in these ratings there was a negative bias towards both patients from Ghana and from Serbia.

3 Bias against patients who are seen as contributing to their injury initially seems to be an odd category compared to the more familiar ones of race and gender. Clinicians may treat brain injured patients differently if they are somehow seen as ‘responsible’ for their injury, for instance, if they were engaging in risk-taking behaviour such as drug taking. Our review was intended to capture studies such as these that identify biases that are specific to clinical contexts and thus of particular interest to clinicians.
